# Excess Air Ratio Management in a Diesel Engine with Exhaust Backpressure Compensation

**DOI:** 10.3390/s20226701

**Published:** 2020-11-23

**Authors:** Piotr Kasprzyk, Jacek Hunicz, Arkadiusz Rybak, Michał S. Gęca, Maciej Mikulski

**Affiliations:** 1 Faculty of Mechanical Engineering, Lublin University of Technology, 20-618 Lublin, Poland; p.kasprzyk@pollub.pl (P.K.); a.rybak@pollub.pl (A.R.); michal.geca@pollub.pl (M.S.G.); 2 School of Technology and Innovations, Energy Technology, University of Vaasa, 65200 Vaasa, Finland; maciej.mikulski@uwasa.fi

**Keywords:** wideband oxygen sensor, exhaust backpressure, pressure compensation, diesel engine

## Abstract

The paper investigates the operation of a wideband universal exhaust gas oxygen (UEGO) sensor in a diesel engine under elevated exhaust backpressure. Although UEGO sensors provide the excess air ratio feedback signal primarily in spark ignition engines, they are also used in diesel engines to facilitate low-emission combustion. The excess air signal is used as an input for the fuel mass observer, as well as to run the engine in the low-emission regime and enable smokeless acceleration. To ensure a short response time and individual cylinder control, the UEGO sensor can be installed upstream of a turbocharger; however, this means that the exhaust gas pressure affects the measured oxygen concentration. Therefore, this study determines the sensor’s sensitivity to the exhaust pressure under typical conditions for lean burn low-emission diesel engines. Identification experiments are carried out on a supercharged single-cylinder diesel engine with an exhaust system mimicking the operation of the turbocharger. The apparent excess air measured with the UEGO sensor is compared to that obtained in a detailed exhaust gas analysis. The comparison of reference and apparent signals shows that the pressure compensation correlations used in gasoline engines do not provide the correct values for diesel engine conditions. Therefore, based on the data analysis, a new empirical formula is proposed, for which the suitability for lean burn diesel engines is verified.

## 1. Introduction

The development of a wideband universal exhaust gas oxygen (UEGO) sensor has extended the conventional application of excess air ratio (λ) signal in engine performance and emission control. The measurement principle is based on the diffusion of gasses between the so-called reference cell and pump cell governed by the yttrium-stabilized zirconia (YSZ) O_2_^−^-conductive membrane [1]. In general terms, this diffusion rate and direction are sensitive to exhaust gas composition; hence, wideband sensors—unlike their discrete-state predecessors—enable the measurement of λ in the whole applicable range and with fast response. 

Owing to their characteristics, combined with high rigidity and stability in corrosive environments, UEGO sensors are now used as a standard control feature for spark ignition engines. In the stoichiometric combustion concept, the main benefit of λ control comes through improved conversion for a three-way catalytic converter [2]. At the same time, the other downstream sensor provides catalytic converter diagnostic and regeneration functionalities [2]. In 2001, Delphi (Delphi Technologies Ltd., London, UK) introduced a production-feasible, wide-range λ control system for gasoline direct injection engines that was based on the wideband UEGO sensor technology [3]. This enabled strict NO_X_ emission limits with reduced calibration burden under lean combustion conditions. Currently, wideband UEGO sensors are also used in diesel engines to help reduce engine-out emissions. The λ signal is used as an input for the fuel mass observer [2], as well as to run the engine in the low-emission area of the map and enable smokeless acceleration. Furthermore, in lean-burn marine gas engines, the absolute λ signal can be used for virtual knock estimation. This wide spectrum of UEGO sensor applications is complemented by virtual λ-based fuel composition sensing for gasoline-ethanol [4] or diesel–FAME (fatty acid methyl ester) [5] flex-fuel engines. 

Putting the engine type aside, more recent λ measurement applications focus on individual cylinder control [6,7]. In such applications, the market-available sensors pose some problems related to feedback response delay. These problems can be overcome by the application of ion sensing at individual cylinders [8]. This real-time λ determination method ensures sufficient accuracy (errors typically do not exceed 3%) [9], while the use of the same hardware offers the additional advantage of combustion timing determination [10]. For UEGO sensors, the relatively long feedback delay problem is usually solved by various adaptive compensation algorithms incorporating Smith predictors [11,12] or other feed-forward/feed-back systems [13]. Furthermore, individual cylinder λ control can be realized with a single UEGO sensor located at the common exhaust runner, while signal delay is dealt with by means of simplified gas transport and mixing models [14]. Finally, it should be noted that completely virtual λ estimation techniques are explored, too [15,16]. These are usually based on fast-running mean value air-path models, which makes them potentially suitable for relatively simple engine layouts without exhaust gas recirculation (EGR), where pulsations dynamics can be neglected.

Still, the robustness and low production costs put the UEGO sensors in the mainstream of on-board λ sensing technologies. However, the aforementioned developments in λ-based control pose additional challenges for sensor accuracy, especially when non-standard test conditions are concerned. The type of fuel may affect oxygen sensor readings. The effect is straightforward for oxygenated fuels and enables on-board FAME admixture estimation [5]. The Bosch (Robert Bosch GmbH, Stuttgart, Germany) R&D group reported significant differences between the readings of their sensors for gasoline and ethanol [4]. On the other hand, Irimescu [17] used the above fuels and observed only minor differences solely in highly lean or highly rich mixture conditions and elevated pressures. Finally, Grannell et al. [18] explained the significant delta in the sensor readings for gasoline and ammonia (NH_3_) combustion by attributing it to the oxidation of unburned NH_3_ on the sensor surface.

The above partially conflicting reports may result from the employment of different sensor technologies or different thermodynamic conditions of measurements. On the hardware side, the sensors usually differ in the reference pumping current definition that is either adjusted to ambient air (e.g., LSU 4.2 sensor by Bosch) or selected arbitrarily (e.g., LSU 4.9 sensor by Bosch) [17]. As far as test conditions are concerned, the diffusion-based operating principle makes sensors very sensitive to the exhaust backpressure [17]. While this is typically not an issue for λ measurement in conventional spark ignition or diesel engine applications, the cylinder-individual estimation methods usually rely on upstream sensor mounting that is subjected to significant pressure fluctuations. These may arise from cycle-to-cycle pulsations in the manifold or, on a longer-time basis, from (operating) point-to-point differences in pre-turbine pressure. Some UEGO sensors available on the market are already equipped with additional exhaust pressure measurement channels and corresponding compensation algorithms [19,20]. However, the nature of these correlations is either not revealed or revealed in a relatively simple form, without taking account of additional aspects such as signal delay or compositional and thermal effects. Regarding the thermal sensitivity of sensor signal, this aspect is important during engine start-up and fast transients. Some fundamental insight into the issue can be found in the work by Harris and Collings [1].

Concluding the above literature review, it should be noted that although the technology has been available on the market for some time, new developments in control put additional constraints on the UEGO sensor signal quality. Consequently, most research in the field focuses on understanding the cross-linking effects of various parameters under non-standard sensor application conditions. The present work fills the knowledge gap in this research by identifying the mechanisms of wideband UEGO sensor signal compensation under heavily lean conditions, high overall fuel dilution rates (heavy external EGR), and excessive exhaust backpressures. The new model is proposed that correlates the ambient-level UEGO signal with exhaust backpressure to provide accurate lambda estimation for a wide range of mixture strengths typical for contemporary lean-burn combustion engines. This correlation is validated under steady-state conditions using the reference λ value calculated from the carbon balance method. The new model surpasses the earlier-proposed UEGO signal pressure compensations in terms of the range of applicability while providing satisfying accuracy across all tested operating conditions. The work is considered instrumental for prospective cylinder-individual λ control strategies.

## 2. Theoretical Background and Definitions

### 2.1. Combustible Mixture and Exhaust Gas Composition

All available engine fuels, fossil and renewable alike, are primarily composed of hydrogen and carbon. Alcohols, ethers, and carboxylic acids additionally contain large amounts of oxygen. Other components, such as inert nitrogen or sulfur, occur in automotive fuels in low concentrations.

Combustible mixture composition in a reciprocating engine cylinder is defined by two main parameters: λ and EGR ratio. The λ is defined as the ratio of the actual air–fuel ratio to the stoichiometric air requirement. The EGR ratio is the fraction of recirculated exhaust gas in the in-cylinder charge. It should be noted that, typically, EGR does not affect the *λ* value because, due to the elemental composition, it is the same for combustible mixture and exhaust gas. Hence, the balance between the main reactants and combustion products on a molar basis can be described by the following equation:

(1) $$C_{m}H_{n}O_{p}+\lambda \left(m+0.25n-0.5p\right)\left(O_{2}+3.76N_{2}\right)\to \;\to {\mathrm{mCO}}_{2}+0.5{\mathrm{nH}}_{2}O+\left(\lambda -1\right)\left(m+0.25n-0.5p\right)O_{2}+3.76\lambda \left(m+0.25n-0.5p\right)N_{2}$$

where m, n, and p denote the numbers of atoms in the generalized fuel molecule $C_{m}H_{n}O_{p}$. It should be noted that exhaust toxic gaseous compounds are not considered in Equation (1); however their concentrations in diesel engines are low and cumulatively do not exceed 1% on a molar basis [21].

### 2.2. Measurement Principles and Calculation Procedures for UEGO Sensor

The UEGO sensor determines the value of λ via the indirect measurement of oxygen concentration in the exhaust gas. The measurement principle is based on the Nernst equation, which relates the reduction potential of an electrochemical reaction in a cell (E) to the standard potential of the electrode (E^0^), gas temperature in the cell, and activities of the chemical species undergoing reduction and oxidation (a_red_ and a_ox,_ respectively) [22]:

(2) $$E=E^{0}+\frac{\mathrm{RT}}{\mathrm{zF}}\ln\left(\frac{a_{\mathrm{ox}}}{a_{\mathrm{red}}}\right).$$

In Equation (2), R and F denote the universal gas constant and the Faraday constant, respectively, and z is the number of electrons transferred in the cell reaction. It should be noted that the activities a_red_ and a_ox_ are always determined with respect to the standard state (1 mol/L for solutes, 1atm for gases), similarly to the reaction equilibrium constants. Therefore, the activities can be narrowed down to respective species concentrations. For practical applications, the concentrations can be further determined via partial pressures of individual species using Dalton’s law—Equation (3). Considering that the ion carrier is oxygen, the voltage of the *λ* transducer can be expressed as:

(3) $$V=t_{\mathrm{ion}}\frac{\mathrm{RT}}{4F}\ln\left(\frac{p_{O_{2}\;\mathrm{air}}}{p_{O_{2}\;\mathrm{exh}}}\right),$$

where t_ion_ is the ionic transference number, and $p_{O_{2}\;\mathrm{air}}$, $p_{O_{2}\;\mathrm{exh}}$ denote the partial pressures of oxygen in air and exhaust, accordingly. 

**Figure 1:**
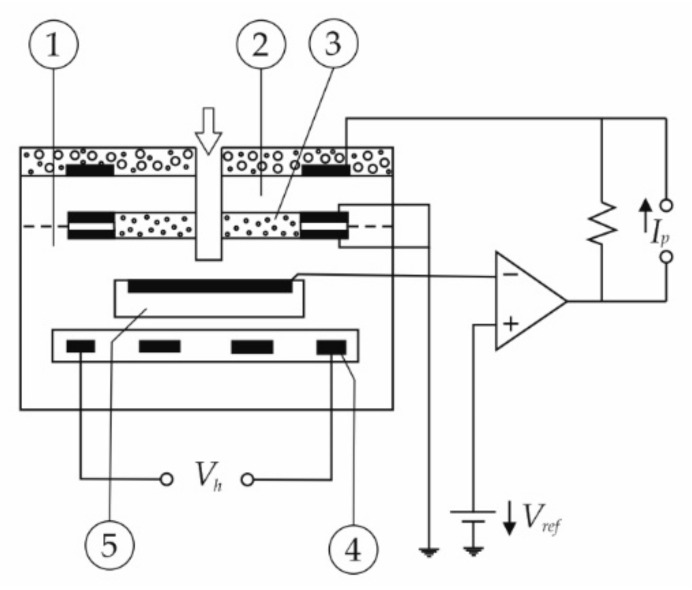
Schematic diagram of a wideband universal exhaust gas oxygen (UEGO) sensor: (**1**) sensor cell; (**2**) pump cell; (**3**) diffusion barrier; (**4**) heater; (**5**) reference air.

To enable wideband operation, the UEGO sensor considered in this study has three measurement chambers that are shown in Figure 1 and referred to as (1) a sensor cell, (2) a pump cell, and (5) a reference air channel.

**Figure 2:**
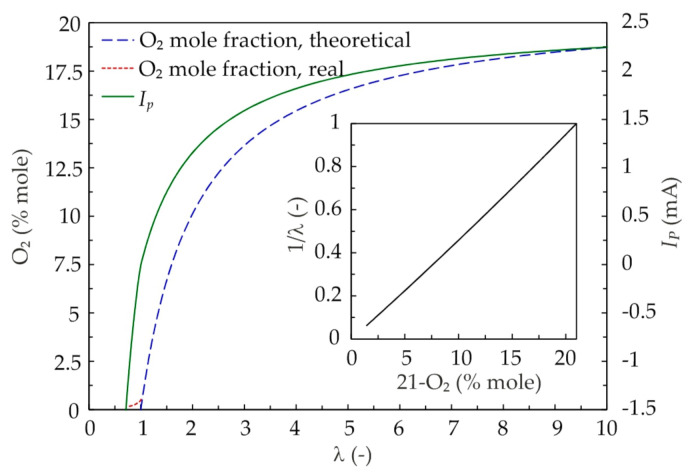
Characteristics of a wide-band UEGO sensor. Theoretical O_2_ mole fraction calculated using Equation (1). Real O_2_ mole fraction from technical report [23]. Pump current from ETAS (ETAS GmbH, Stuttgart, Germany) LA4 lambda meter manual [24].

The sensor cell is in contact with exhaust gases on one side and is flushed by air on the other. The voltage produced by the sensor’s Nernst cell is compared to the set value of 0.45 V, which corresponds to the stoichiometric mixture. Any deviation from this value switches the pump current that controls the activity of the oxygen pump. Oxygen from the exhaust gas is pumped outside or inside, so that the voltage in the measuring chamber is maintained constant at 0.45 V, which provides the stoichiometric exhaust in the measuring cell. The pumping current (I_p_) required to sustain this state is directly measured. It correlates nearly linearly with the λ value of the fuel–air mixture. The response of the complete lambda measurement system is shown in Figure 2, including the relation of the pumping current to λ and the associated O_2_ mole fraction. By calibrating the pump current to the oxygen concentration resulting from Equation (1), the characteristics of the measurement system can be linearized, as shown in a subplot in Figure 2.

It should be noted that the real O_2_ mole fraction in the exhaust gas (red dashed line in Figure 2) differs from the theoretical one determined from Equation (1) (blue dashed line in Figure 2) due to an incomplete utilization of oxygen. In such case, a small amount of oxygen remains even under rich mixture. The difference is mainly manifested in the rich mixture range (λ < 1), where the emissions of unburned hydrocarbons (HC) and carbon monoxide (CO) influencing the molar balance in Equation (1) become excessive. In the range of lean mixtures, which is the case for diesel engines, the difference between real and theoretical oxygen concentrations is small. Additionally, the combustion efficiency in diesel engines is much higher than in spark ignition engines, thus resulting in lower CO and HC emissions.

The above considerations are valid for ambient pressure conditions denoted here as *p*_0_. It should be observed that at a given mole fraction, the partial oxygen pressure is proportional to the gas pressure; thus, the UEGO sensor should respond to the pressure change similarly to the λ change. The effect of exhaust backpressure on the pump current can be described using the following empirical formulation from the Bosch LSU 4.2 Technical Customer Information [25]:

(4) $$I_{P\_\mathrm{app}}=I_{P}\left(p_{0}\right)\frac{p_{\mathrm{exh}}}{k+p_{\mathrm{exh}}}\cdot \frac{k+p_{0}}{p_{0}},$$

where p_exh_ denotes the exhaust pressure and I_P_(p_0_) is the pump current at ambient conditions. The k factor for lean mixtures was experimentally determined as 47 kPa. Taking another model from [17] and performing some manipulations, it is possible to directly estimate the apparent value of λ, as expressed by Equation (5):

(5) $$\lambda_{\mathrm{app}}=\frac{\lambda \left(p_{0}\right)}{1+m\cdot \Delta p\left[1-\lambda \left(p_{0}\right)\right]}\;,$$

where Δp = p_exh_ − p_0_. The constant *m* was experimentally determined to be 6.25·10^−3^ kPa^−1^. It should be noted that both above-mentioned correlations were validated only for nearly stoichiometric conditions (1 ≤ λ ≤ 1.2), which is relevant for homogeneous gasoline engine operation. In the case of diesel engines, the behavior of the sensor would be significantly different at high excess air conditions. The present research fills this knowledge gap, and the validity of Equations (4) and (5) is critically assessed in the Discussion section.

## 3. Methods

### 3.1. Test Stand

The experiments were performed using a single-cylinder research engine type 5402 CRDI by AVL (AVL List GmbH, Graz, Austria). To enable the simulation of engine operation under normal service conditions, the engine was coupled with the AC dynamometer with advanced automatic control. The use of the single-cylinder engine provided high exhaust pulsations that occur when the cylinder-individual λ control is applied.

Detailed specifications of the test engine are given in Table 1.

For the purpose of this study, it suffices to mention that the test platform had a compression ignition-based combustion system with a toroidal in-piston combustion chamber coupled with a seven-hole electromagnetic injector representing the state-of-the-art light-duty automotive diesel engine.

**Figure 3:**
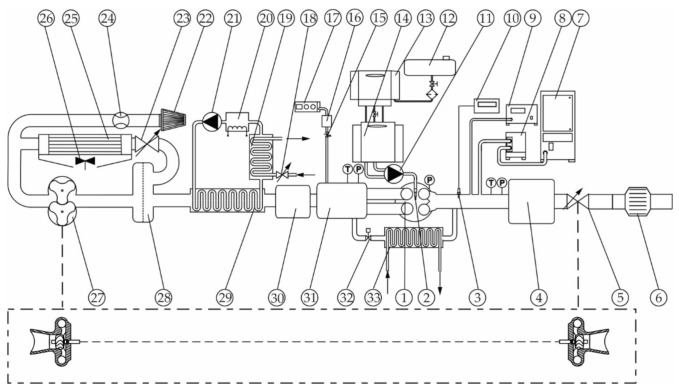
Schematic diagram of the engine test stand: (**1**) engine; (**2**) fuel injector; (**3**) UEGO sensor, (**4**) exhaust plenum; (**5**) exhaust backpressure valve; (**6**) exhaust muffler; (**7**) Fourier Transform Infrared (FTIR) analyzer; (**8**) heated filter; (**9**) soot meter; (**10**) LA4 lambda meter; (**11**) high pressure fuel pump; (**12**) fuel tank; (**13**) fuel consumption meter; (**14**) fuel conditioner; (**15**) intake air control valve; (**16**) intake air plenum chamber; (**17**) intake gas analyzer; (**18**) intake cooling valve; (**19**) heat exchanger; (**20**) air coolant electric heater; (**21**) air coolant pump; (**22**) air filter; (**23**) bypass valve; (**24**) air-flow meter; (**25**) bypass air cooler; (**26**) air cooler fan; (**27**) roots compressor; (**28**) oil separator; (**29**) intake air heat exchanger; (**30**) intake plenum; (**31**) intake plenum/exhaust gas recirculation(EGR) mixing chamber; (**32**) EGR control valve; (**33**) EGR cooler.

Injection parameters were managed by a fully opened engine control unit, ETAS INCA. During the experiments, the engine was operated as both naturally aspirated and supercharged in order to enable a high range of excess air at variable exhaust EGR rates. The cooled external EGR delivery rate was controlled via a proportional valve. A mechanical roots compressor driven by an electric motor was used to ensure flexible control of boost pressure. For achieving high EGR rates at boost conditions, an exhaust backpressure valve was installed downstream of the exhaust plenum. All components of the air-path system used in the experiments are shown in Figure 3.

The wideband UEGO sensor used for providing the apparent λ signal (λ_app_) was a Bosch LSU 4.2 connected to the ETAS (ETAS GmbH, Stuttgart, Germany) LA4 lambda meter. The sensor was installed in a straight exhaust runner, approximately 200 mm away from the exhaust valves. The reference λ value was determined with the use of the AVL (AVL List GmbH, Graz, Austria) SESAM FTIR (Fourier Transform Infrared) multi-component gas analytical system. Additionally, the Pierburg–Hermann (Hermann Electronic, Fürth, Germany) HGA400 gas analyzer was used to measure the intake CO_2_ concentration and thus to provide estimation of the EGR rate.

The engine test stand was equipped with all other measurement devices required for completion of the planned tests. Among others, the measurement system consisted of an intake air thermal mass flow meter, a precision fuel balance with thermal conditioning, and a set of pressure and temperature transducers for measuring intake and exhaust thermodynamic conditions. The intake air temperature as well as the cooling agent and lube oil temperatures were controlled via separate thermal management systems. Further details of the experimental apparatus are provided in Appendix A.

### 3.2. Experimental Procedure

**Figure 4:**
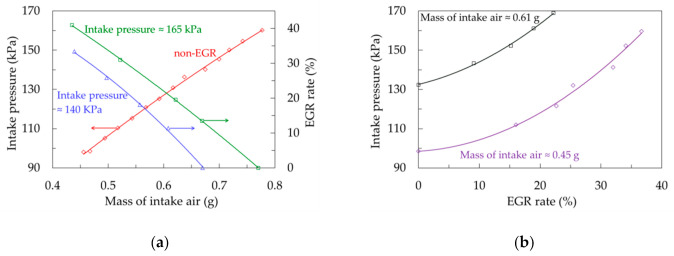
Explored intake gas conditions relevant to UEGO signal quality: (**a**) EGR sweeps at constant pressure and pressure sweep at non-EGR; (**b**) intake pressure and EGR relations at constant amounts of in-cylinder air.

The engine was fueled with commercial (EN 590 standard) diesel fuel. To avoid the impact of variable exhaust runner dynamic pressure changes on λ readings, the experiments were conducted at a constant rotational speed of the engine (2000 rev/min), but at variable fueling, boost pressures, and EGR rates. The exact parameter sweeps are not discussed in detail here because their sole purpose was to provide the exhaust gas with the required air excess and pressure. Therefore, it suffices to mention that the applied engine settings replicated a wide range of real engine operating envelopes extrapolated toward ultra-lean operation and heavy EGR conditions. Figure 4 shows the relations between gas exchange control parameters.

At a fueling rate of approximately 15 mg/cycle, the presented range of intake air mass provided the λ span ranging from 1.95 to 3.5. With maximum attainable boost pressure of 1.6 bar, further enleanment of the mixture was achieved by reducing the fueling rate. The complete experimental matrix included variations of mass of fuel injected from 6 to 16 mg, providing a brake mean effective pressure range from 0 to 0.5 MPa. Under all operating conditions, the exhaust backpressure was maintained at a level reflecting the turbocharger coupling.

Additionally, it is worth noting that under all tested conditions, the engine injection control parameters were adjusted to avoid excessive exhaust emissions, as they could affect λ readings (refer to Section 2.1 and Section 2.2. For details). To this end, fuel was injected using the split injection technique, which provides the best soot (particulates)/NO_X_ trade-off (constant main fuel injection at 8 Crank angle degrees before the top dead center; 1.4 mg pilot fuel value with pilot injection timing optimized). At neither of the operating points, the NO_X_ or particulate matter emissions did exceed 4.5 or 0.3 g/kWh, respectively. At the same time, CO and HC emissions were constrained below 2 and 1 g/kWh, limiting the EGR/λ exploration space to values below 40% and above 1.4, respectively. Further data analysis relied on comparing the λ value measured directly using the UEGO sensor and dedicated signal conditioner (LA4) with the λ calculated by the carbon balance equation with FTIR readings as inputs. The AVL SESAM FTIR enables the simultaneous measurement of the content of specific hydrocarbons, nitrogen oxides, and other chemical exhaust gases compounds. The exhaust gases were transferred from the engine to the FTIR analyzer through a heated line. The response time of the analyzer was a single second. The time-averaged value of the 30 s measurement period was taken as a single reading result. The reference value calculation procedure is discussed in detail below.

### 3.3. Reference λ Determination

The reference λ value (λ_ref_), considered as the real value, was calculated using the carbon balance in Equation (6) for wet exhaust analysis [26] in the following way

(6) $$\lambda_{\mathrm{ref}}={\left(\frac{m_{\mathrm{fuel}}}{m_{\mathrm{air}}}\right)}_{\mathrm{stoi}}\frac{M_{\mathrm{air}}}{M_{\mathrm{fuel}}}\left(\frac{1+x_{\mathrm{HC}}-0.5x_{\mathrm{CO}}+0.5x_{H_{2}O}}{x_{\mathrm{HC}}+x_{\mathrm{CO}}+x_{{\mathrm{CO}}_{2}}}-0.5y\right).$$

The results of exhaust composition from the FTIR multi-compound analytical system served as an input for this equation. The *x* with respective subscript denotes the concentration of total HCs, CO, CO_2_, and H_2_O in the exhaust at a given operating point. M_air_ is the average molecular mass of air, M_fuel_ is the molecular mass of the fuel, while (m_fuel_/m_air_) stoi is the stoichiometric fuel–air ratio, i.e., the theoretical air demand. The molecular ratio of hydrogen to carbon (*y*) for the diesel fuel used in the present research was 1.875.

It should be noted that the method does not rely on the O_2_ concentration. More importantly, since the λ obtained via Equation (6) is directly composition-based, it is insensitive to changes in the measurement conditions (pressure, temperature, EGR, etc.). Hence, the method discussed here provides a reliable reference value for validating measurement results from the UEGO sensor.

## 4. Results

**Figure 5:**
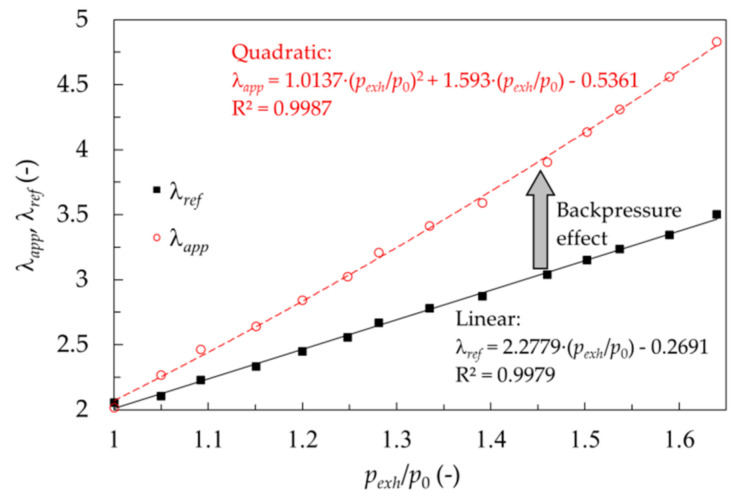
Comparison of λ_app_ and λ_ref_ for non-EGR conditions, constant fueling rate of 15 mg/cycle.

As highlighted in the introduction and elaborated on in the background section, the UEGO sensor readings depend on the pressure in the measurement environment. Figure 5 confirms this thesis by comparing the λ signal from the Bosch LSU 4.2 UEGO sensor (λ_app_) with the λ results obtained via FTIR measurements and Equation (6) (λ_ref_) at a variable exhaust-to-ambient-pressure ratio.

At the 101 kPa exhaust pressure, the λ values obtained with the Bosch sensor and FTIR analyzer agree. At constant fueling, the FTIR results representing the real value are linear to the exhaust pressure because the exhaust pressure followed the intake pressure, which nearly linearly translates to cylinder volumetric efficiency. Due to the UEGO sensor measurement principle, an increase in the exhaust pressure results in the overestimation of λ_app_. At a given oxygen mole fraction, the partial pressure is proportional to the exhaust pressure, thus changing the difference in oxygen concentrations on opposite sides of the membrane. It should be noted that reference air is under ambient pressure. In turn, this force increased pump operation to equalize the above concentrations (voltages). As the pump current is converted to lambda, the effect of pressure is nearly quadratic, as shown in Figure 5. As a result, for the highest tested pressure ratio of 1.64, the lambda sensor overestimated the actual lambda value by over 37% (λ_app_ = 4.81 vs. λ_ref_= 3.49). Note the measurement points presented in Figure 5 are time-averaged values (sampled at 1 Hz over 60 s acquisition window) from individual steady-state operating points. For the discussed no-EGR conditions, the instantaneous readings of both the Bosch LSU 4.2 UEGO sensor (λ_app_) and the exhaust pressure probe were very stable pertaining to the high fidelity of thermal management used in our single cylinder research engine. The standard deviations of λ_app_ and p_exh_/p_0_ did not exceed 0.1 and 0.02 respectively, for all points presented on the red curve. The diameter of the red circles in Figure 5 gives a good estimate of maximum uncertainty here. The standard deviations of λ_ref_ were not available because the instantaneous compositions in Equation (6) were not recorded.

**Figure 6:**
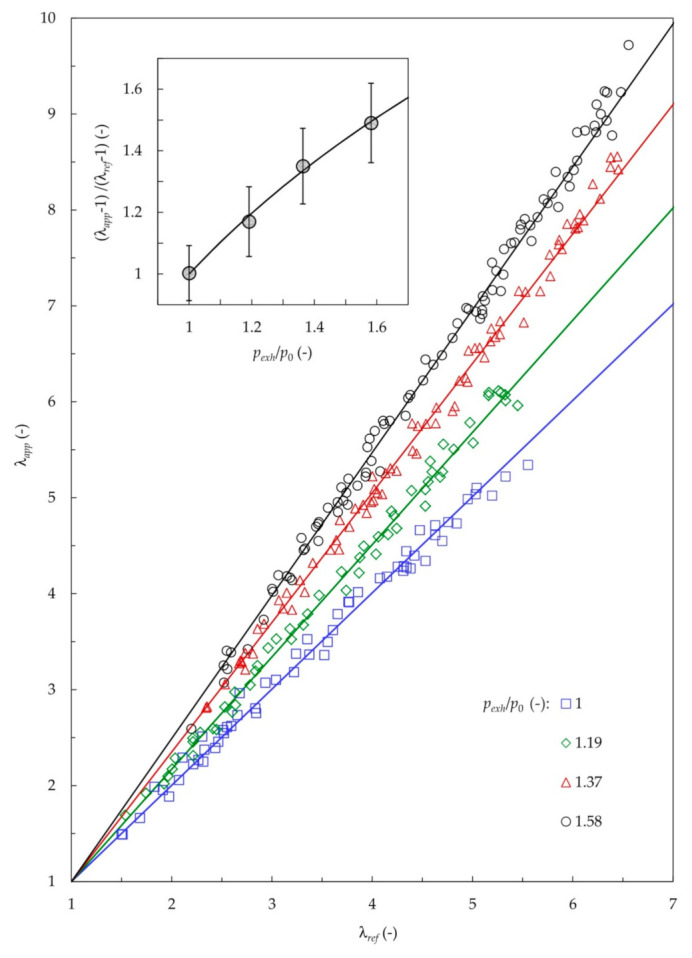
λ_app_ vs. λ_ref_ for different exhaust pressures. The graph inside presents the directional coefficient of linear fit, where error bars denote standard deviations of the residual component.

Figure 6 shows the cumulative results of different EGR range sand fueling rates grouped by color-coding into four exhaust pressure ranges.

It can be noted that for the exhaust pressure at ambient pressure, there is a good agreement between the results obtained with the UEGO sensor and the reference carbon balance method. For the individual exhaust pressure, the correlation between the two signals is linear. It should be noted that the dispersion of the measurement points around the line of regression increases with increasing λ. This directly results from the decreasing sensitivity of the sensor (see Figure 2 for reference), rather than from the EGR itself. No EGR dependence is observed between the results, and the overall dispersion around the respective exhaust pressure fits is the combined effect of measurement accuracy and other factors such as sampling temperature. 

In addition to the above, it should be noted that the exhaust pressure itself was controlled by the system with an accuracy of ±2 kPa. According to the dependencies established in the present study, this translates to roughly 1.23% of the possible error caused solely by a point-to-point deviation in the exhaust pressure.

The pressure ratio is of prime significance. Since λ_app_ changes linearly with λ_ref_ for individual pressure ratios, the directional coefficient of this line changes with the pressure ratio itself. This dependence is plotted in the upper left corner of Figure 6. One can note that the relation here is logarithmic and can be explicitly formulated as shown in Equation (7):

(7) $$\frac{\lambda_{\mathrm{app}}-1}{\lambda_{\mathrm{ref}}-1}=1.08\cdot \ln\left(\frac{p_{\mathrm{exh}}}{p_{0}}\right)+1.$$

Adopting the nomenclature of the previous UEGO sensor models discussed in Section 3.3, Equation (7) can be used to model the output of the sensor (λ_app_) at different exhaust backpressure values. Assuming that the value of λ_ref_ is always equal to λ(p_0_), one gets

(8) $$\lambda_{\mathrm{app}}=\left[\lambda \left(p_{0}\right)-1\right]\left[k\cdot \ln\left(\frac{p_{\mathrm{exh}}}{p_{0}}\right)+1\right]+1$$

where k = 1.08. However, it should be stressed that the above equation is only valid for lean mixtures.

## 5. Discussion and Outlook

**Figure 7:**
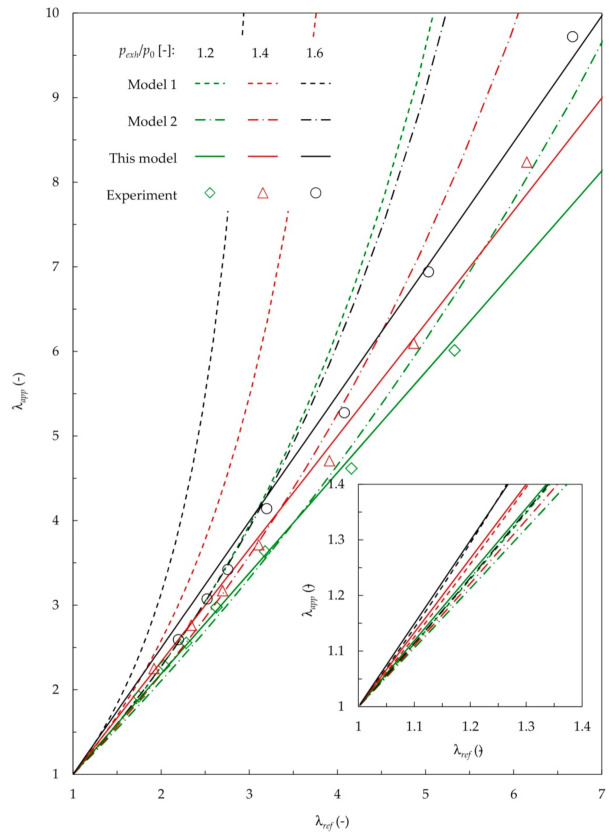
Comparison of λ_app_ calculated using available models (Model 1—Equation (5) [17], Model 2—Equation (4) [25]) and the model elaborated in this study with experimental verification data.

This section benchmarks the wideband lambda sensor model proposed in Equation (8) against other correlations available in the literature. Model 1 refers to the correlation by Irimescu [17] expressed directly through Equation (5). Model 2 refers to the approach by Bosch [25], where the sensor characteristics from Figure 2 were used to convert the pump current expressed by Equation (4) to the λ_app_ values. Figure 7 presents the results of this benchmark.

According to the results, the two previous UEGO models proposed for turbocharged spark ignition engines, i.e., Model 1 and Model 2, fail under large excess air conditions that are typical of the diesel engine operation regime. On the other hand, the new model shows a good agreement with the experimental findings in a wide range of λ values between 2 and 7. The maximum relative error did not exceed 7%. 

It is worth emphasizing that reliable validation results for Model 1, covering the λ range from 1 to 1.2, were presented in Irimescu [17]. However, due to the diesel combustion regime restrictions, this region was beyond the scope of the present study. Still, the model proposed in this work makes it possible to capture the low lambda range with good accuracy, as shown by the inner graph plotted in Figure 7. The deviation from Model 1 in this range did not exceed 0.8%. On the other hand, compared to the experimental findings of this study, the error of Model 1 at λ = 2 was 3.5%, 9.5%, and 20% for the exhaust-to-ambient-pressure ratios of 1.2, 1.4, and 1.6., respectively. Ultimately, the higher the backpressure, the higher the λ estimation error becomes for Model 1 and Model 2 alike. However, this is not the case with the new model, where the probability distribution is relatively independent of the backpressure. 

Finally, it should be noted that the Model 2 predictions appear to fit the results better than those obtained with Model 1 at a larger range of lambda values. Nevertheless, it must be observed that the lambda results obtained with Model 2 are drastically underestimated for the range of its intended usage. For λ between 1.2 and 1.4, the discrepancy between the predictions of Model 1 (carefully validated results are available) and Model 2 amounts to 11%. It should be observed that Model 2 is typically used for UEGO sensor calibration; however, its validation results have not been revealed. The present work suggests that the suitability of Model 2 is questionable, even for typical spark ignition engine applications.

The new model derived in this work is instrumental for cylinder-individual lambda control strategies enabling the next generation of lean-burn engines. The potential application ranges from incremental improvement of conventional diesel combustion (to improve cold-start strategies with smokeless combustion) to advanced low-temperature combustion concepts such as HCCI or RCCI. In the latter case, the λ management issue is of prime importance for achieving controllable combustion with superior efficiency and emission trade-off [27,28].

The following steps form our research agenda in this direction: (1) in the short term, the new sensor model will be used to develop a real-time-capable correction routine and to test it in transient engine operation together with a response delay correction routine; (2) in the long term, the advantage of individual cylinder-based λ control will be demonstrated using the advanced low-temperature combustion platform developed by the authors in [29].

## 6. Conclusions

The results of the present study lead to the following conclusions:

- The exhaust pressure has a pronounced effect on the UEGO sensor output. With the exhaust pressure increased to 160 kPa, the overestimation of λ by the UEGO sensor may be as high as 37%;
- At the same time, the exhaust pressure has a more significant influence on the sensor output than all other factors combined, including exhaust thermal conditions, EGR, and cumulative measurement accuracy;
- The previous linear models for wideband UEGO sensor response to backpressure proposed for spark ignition engine applications fail drastically for diesel-like excess air ratios. Whenλ ≈ 2, the error can be as high as 20% and may increase to over 65% for λ higher than 3;
- The proposed new model has a minimum accuracy of 7% across the wide spectrum of λ values (from 1.4 to 7) and EGR (0–40%), as investigated in this study;
- The comparison with an accurately validated spark ignition-capable model (Model 1) has shown that the proposed model yields a satisfactory accuracy of 0.8% at 1 ≤ λ ≤ 1.2.

## Figures and Tables

**Table 1:** Research engine specifications.

Type	AVL 5402
Configuration	Four-stroke, single-cylinder
Bore	85 mm
Stroke	90 mm
Displacement	510.5 cm^3^
Compression ratio	17:1
No. of valves	4
Combustion type	Direct injection
Max. fuel pressure	180 MPa
Injection system	Common Rail CP4.1
Engine management	AVL-RPEMS, ETK7-Bosch

**Figure A1:**
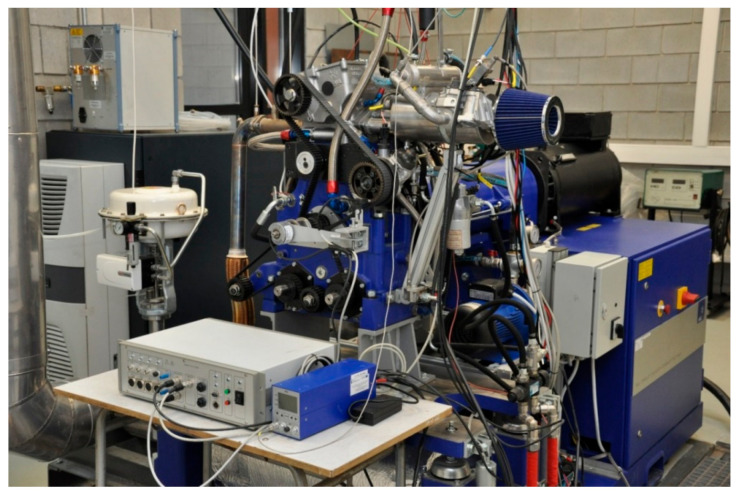
Overall view of the 5402 AVL engine test stand.

**Figure A2:**
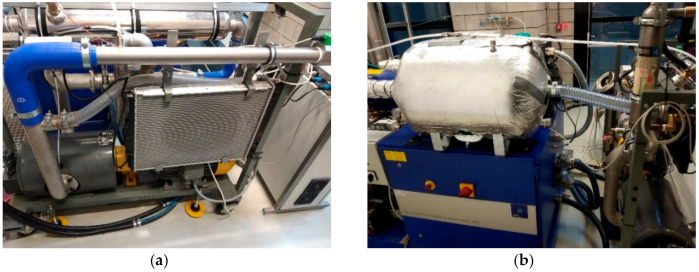
The intake air preparation system; (**a**) airflow meter, supercharger and the charge air cooler; (**b**) pulsation-reducing intake plenum.

**Figure A3:**
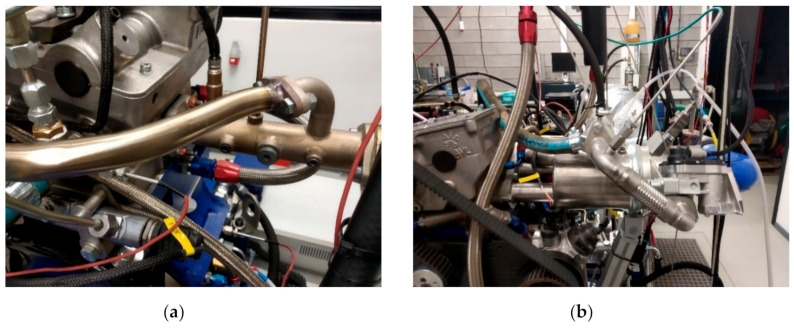
The intake and exhaust engine runners; (**a**) exhaust runner with UEGO sensor, exhaust pressure sensor, and EGR runner system; (**b**) EGR mixing chamber with sensors and temperature conditioning system (on the right).

## Abbreviations

**List of acronyms**	
CO	carbon oxide
CRDI	common rail direct injection
EGR	exhaust gas recirculation
FAME	fatty acid methyl ester
FTIR	Fourier transform infrared
HC	hydrocarbons
HCCI	homogenous charge compression ignition
NO_x_	nitrous oxides
NH_3_	ammonia
RCCI	reactivity-controlled compression ignition
UEGO	universal exhaust gas oxygen sensor
YSZ	yttrium-stabilized zirconia
**List of symbols**	
a_ox_	species undergoing oxidation (1 mol/L) for solutes, (1 atm) for gases
a_red_	species undergoing reduction (1 mol/L) for solutes, (1 atm) for gases
E	potential of an electrochemical reaction in a cell (V)
E^0^	standard potential of electrode (V)
F	Faraday constant (C/mol)
I_P_app_	pump current under exhaust overpressure (A)
I_P_(p_0_)	pump current at ambient conditions (A)
k	experimentally determined factor for lean mixtures (-)
p_0_	ambient pressure (MPa)
p_exh_	exhaust pressure (MPa)
p_O_2_ air_	partial pressure of oxygen in air (MPa)
p_O_2_ exh_	partial pressure of oxygen in exhaust (MPa)
R	universal gas constant (J/mol × K)
T	temperature (K)
t_ion_	ionic transference number (-)
V	voltage of the λ transducer (V)
**Greek letters**	
λ	excess air ratio (-)
λ_app_	apparent lambda value under exhaust overpressure (-)

## Appendix A

This appendix aims to provide more insight into the layout of the sensors used in the present research. The appendix complements the discussion of the engine air-path in the main body of the text (Section 3.1 and Figure 3). The complete AVL 5402 CRDI engine research setup is shown in Figure A1. Elements of the intake air preparation system are shown in Figure A2. In the intake path, the filtered air goes through a flow meter; then, it is compressed by a mechanical charger. A parallel-mounted (passive) charge air cooler lowers the temperature of the air from the supercharger to approximately 20 °C. If necessary, the air can further be heated in the series mounted heater. This device, visible on the right-hand edge of Figure A2a, is feed-forward controlled and is ultimately responsible for keeping the set charge temperature, independently of the engine operating point and ambient conditions. The charge air pressure is also closed-loop controlled via the electric motor driving the intake air charger. The accuracy of both actively controlled variables is ±0.1 °C and ±2 kPa respectively for intake air temperature and pressure.

The intake air is further homogenously mixed with recirculated exhaust gasses in the EGR mixing chamber mounted approximately 120 mm from the intake port. The EGR circuit has a separate thermal management system with a controllable EGR cooler. The EGR mixer (see Figure A3b) is preceded by a larger intake plenum (Figure A2b) that reduces pressure pulsations generated by the compressor.

The UEGO sensor, being the object of the present research, is mounted in a straight runner approximately 200 mm (see Figure A3b) from the exhaust port. This ensures that the flow is stabilized and uniform at the sensor location while. The distance from the exhaust port is at the same time sufficiently small to ensure that the UEGO sensor always reaches its operating temperature (>100 °C), for all possible engine operating points. Similarly to the intake side, an Exhaust plenum is installed to reduce the gas pressure pulsations in the exhaust system. An in-series mounted, adjustable valve simulates the restriction generated in the areal engine by the turbine element. The level of the exhaust backpressure is closed-loop controlled (±2 kPa) with the valve opening position. Controlling of the compressor and the exhaust backpressure valve separately enables the simulation of a wide range of the turbocharger’s operating conditions. Both on the exhaust and the intake side, the temperatures and pressures are measured at multiple locations. The entire engine air-path conditioning system, including all other elements presented in Figure 3, yet not explicitly mentioned in this appendix, allows for precise control of tall flow streams and their thermal conditions. This functionality enabled the present research.
